# Plant phenotype relationship corpus for biomedical relationships between plants and phenotypes

**DOI:** 10.1038/s41597-022-01350-1

**Published:** 2022-05-26

**Authors:** Hyejin Cho, Baeksoo Kim, Wonjun Choi, Doheon Lee, Hyunju Lee

**Affiliations:** 1 School of Electrical Engineering and Computer Science, Gwangju Institute of Science and Technology, Gwangju, 61005 Republic of Korea; 2 Digital Curation Center, Korea Institute of Science and Technology Information, Daejeon, 34141 Republic of Korea; 3 Department of Bio and Brain Engineering, Korea Advanced Institute of Science and Technology, Daejeon, 34141 Republic of Korea

**Keywords:** Data publication and archiving, Data acquisition

## Abstract

Medicinal plants have demonstrated therapeutic potential for applicability for a wide range of observable characteristics in the human body, known as “phenotype,” and have been considered favorably in clinical treatment. With an ever increasing interest in plants, many researchers have attempted to extract meaningful information by identifying relationships between plants and phenotypes from the existing literature. Although natural language processing (NLP) aims to extract useful information from unstructured textual data, there is no appropriate corpus available to train and evaluate the NLP model for plants and phenotypes. Therefore, in the present study, we have presented the plant-phenotype relationship (PPR) corpus, a high-quality resource that supports the development of various NLP fields; it includes information derived from 600 PubMed abstracts corresponding to 5,668 plant and 11,282 phenotype entities, and demonstrates a total of 9,709 relationships. We have also described benchmark results through named entity recognition and relation extraction systems to verify the quality of our data and to show the significant performance of NLP tasks in the PPR test set.

## Abstract (summary)

**Table:** 

Measurement(s)	Report from Literature
Technology Type(s)	manual curation
Sample Characteristic - Organism	Homo sapiens

## Background & Summary

With rapid technological advancements, data from various fields have been accumulated regardless of the domain, and the accumulated text collection contains innovative information that has not been structured thus far^1^. In particular, the biomedical domain contains useful information, such as those pertaining to new drug resources, and many studies continue to be conducted for the collection of meaningful information for the identification and exploration of interesting patterns based on such data sources^2–4^. However, most data remain unorganized because the volume of new data continues to increase faster than our ability to process such data to construct and analyze meaningful information; thus, manual conversion of data into structured forms is impossible^5,6^. Therefore, essential goals of natural language processing (NLP) include the extraction of meaningful data and the construction of significant information from unstructured text in an efficient and accurate manner^7,8^. Currently, the use of deep learning-based techniques has recently ushered impressive improvements in the accuracy of many applications^9^. Additionally, NLP models can be improved by implementing a deep learning approach because many researchers have demonstrated the achievement of state-of-the-art results across many fundamental NLP tasks and the highest scores^10–12^.

In pharmaceutical development, plants are known for their therapeutic potential over thousands of years, indicating the possibility of obtaining a wide range of natural products from trees, shrubs, herbs, and crops with pharmaceutical capabilities^13,14^. Particularly, medicinal plants and plant-derived medicines are widely used as therapeutic agents in traditional cultures and their efficacy can be verified by numerous clinical studies and medical records based on accumulated experience. Although natural compounds within plants may cause grave side effects, many modern pharmacological drugs are derived from medicinal plants as vital resources^15,16^. Owing to such characteristics, many researchers have examined the therapeutic effects of various botanicals by elucidating the mechanisms of action of these plants. As interest in the development of new drugs from medicinal plants has increased, scientific investigations into the beneficial effects of plants include the extraction of various sources of information such as those pertaining to disease prevention and health promotion in addition to clinical treatment; thus, the definition of the entity type that can encompass such sources is necessary. Herein, the term “phenotype” refers to a wide range of characteristics observable in a human. To extract novel information on plants and phenotypes that have not been organized based on the accumulated literature, the NLP technique can be applied using deep learning techniques. In other words, for improvements in deep learning techniques, a free large-scale and well-constructed corpus may play an important role and it reflects the performance of deep learning models.

These issues highlight the necessity of creating a large-scale and high-quality dataset for NLP tasks. All biomedical NLP communities share the features of remarkable efforts into the performance of high-quality manual annotations of biomedical literature. In the biomedical domain, widely used datasets include 2010 i2b2/VA^17^ and ShARe/CLEF^18^ for clinical texts, NCBI disease corpus^19^ for diseases, BioCreative II Gene Mention corpus (BC2GM)^20^ and JNLPBA^21^ for gene and proteins, BioCreative V Chemicals Disease Relationship (BC5CDR)^22^ for diseases and chemicals, CHEMDNER^23^ for drug and chemicals, LINNAEUS^24^ and Species-800^25^ for species, and the Plant corpus^14^ for plant entities. Moreover, several biomedical relation corpora exist for elucidating various relationships between biomedical entities such as AIMed^26^, BioInfer^27^, CHEMPROT^28^, DDI^29^, EU-ADR^30^, GAD^31^, CoMAGC^32^, Plant-Disease^33^, and Plant-Chemical^13^. Most text corpora contain a detailed markup of several types of entities and relationships in a limited number of abstracts or articles but cannot reflect the relationships between plants and phenotypes mentioned in biomedical publications.

Here, we present the plant-phenotype relationship (PPR) corpus, a resource established to support the development and evaluation of various tasks and to extract new information in the biomedical domain. Using the proposed guidelines, we manually annotated 600 abstracts from the PubMed database. In the PPR corpus, 5668 plant and 11,282 phenotype mentions were annotated, and 9709 relationships between them including “Increase,” “Decrease,” “Association,” and “Negative” were annotated. As the PPR corpus is split into three types—train, development, and test sets—we suggest that our corpus will be invaluable for advancing and evaluating text-mining techniques for biomedical NLP tasks. The PPR corpus is publicly available for various studies in the biomedical domain.

## Methods

### Selection of candidate abstracts

To construct the corpus, annotators independently annotated the mentions of plants and phenotypes and their relationships in the given candidate abstracts. In this section, we describe the process of selecting and annotating candidate abstracts.

We first automatically extracted data on 13,408,586 scientific abstracts from the PubMed database using PubTator^34^ with automated annotations from state-of-the-art text mining systems for biomedical entities. PubTator is used along with DNorm^35^ and SR4GN^36^ to pre-tag disease and species names in the articles, respectively. However, these tools are not sufficient for the detection of plant and phenotypic entities. For the annotation of entity names, an entity mention with its offset of the location in texts must be identified. In the case of plants, we utilized LingPipe^37^, a dictionary-based named entity recognition tool, with a plant name dictionary derived from the NCBI Taxonomy database^38^ to pre-annotate plant names in the abstracts. The NCBI Taxonomy dictionary contains 151,250 concepts and 315,173 terms in English, Chinese, and Latin. To improve the accuracy, we removed stopwords from the dictionary, which frequently appear in the texts but are not related to plants, such as anemia (Taxonomy ID: 12939), lens (Taxonomy ID: 3863), laser (Taxonomy ID: 62990), NAME (Taxonomy ID: 55581), and thymus (Taxonomy ID: 49990). For phenotypes, we applied the deep learning named entity recognition (NER) model^39^ trained by using the NCBI disease corpus to extract data on disease terms^19^. We also used MetaMap^40^, a configurable application for mapping biomedical text to the UMLS Metathesaurus, to retrieve information about additional clinical terms. Therefore, we combined the results of both NER models into a pre-annotated phenotype.

**Fig. 1:**
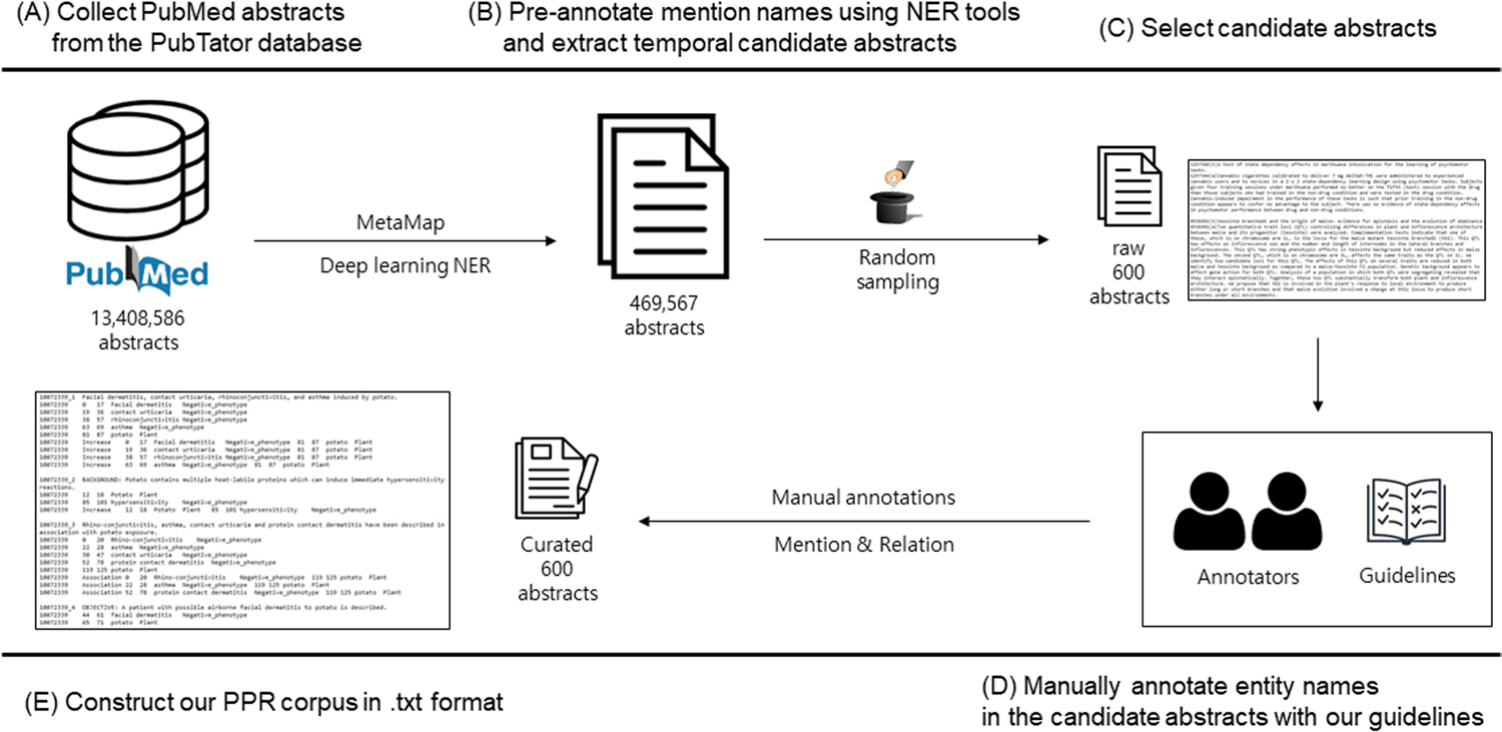
The pipeline of the PPR corpus construction.

Using the pre-annotated mentions of plants and phenotypes, a total of 704,372 co-occurrence sentences from 469,567 pre-annotated abstracts were obtained, in which at least one plant name and phenotype name co-occurred. We then randomly selected 600 candidate abstracts from the pre-annotated abstracts. In spite of pre-processing which helped to remove stopwords for plant names and to include disease NER results, inappropriate entities remained in the candidate abstracts. Therefore, heuristic post-processing with additional annotations is necessary to precisely define the type of plant and phenotype desired. During the annotation task, annotators independently annotated the mentions of plants, phenotypes, and their relationships in the candidate abstracts. Figure 1 depicts the workflow of our corpus construction, and details of the annotation guidelines are described in the next section.

### Annotation guidelines

Annotation guidelines were established to improve inter-annotator agreement for the manual annotation task. The workflow of the PPR corpus construction involved the following two main annotation steps: a mention-level annotation and a relation-level annotation. Here, we describe how annotators annotated mentions and relations from the candidate abstracts. The guidelines include the annotation process of plant and phenotype mentions as the mention-level annotation and highlight the relationships between them as relation-level annotations. Thus, guidelines were categorized for entity annotation and relationship annotation. In the annotation step, the brat rapid annotation tool (BRAT)^41^, an intuitive web-based tool for text annotation supported by natural language processing (NLP) technology, was used to maximize the annotation efficiency.

At this stage, the annotators checked whether the pre-annotated mentions in the abstracts were correctly annotated. If the pre-annotation was incorrectly added due to NER errors, the annotators corrected them to the best of their knowledge. Moreover, most NER systems have been developed based on the sequence labeling method, in which each token is classified as a single label. For this reason, the entity annotations only focus on the longest entity, without the inner nested entities. To maximize the accuracy of the annotations, the annotators formulated guidelines for annotating mentions through the discussion. The annotators followed the guidelines for the annotation task.

#### Annotation of plant mentions

As previously mentioned, plant mentions were first pre-annotated using LingPipe with the NCBI Taxonomy dictionary. Details regarding the guidelines for the annotation of plant mentions are as follows.

- Annotators manually annotate plant names based on the pre-annotated mentions. In this step, the NCBI taxonomy dictionary was used to annotate the exact plant names.
- Annotators should annotate all synonyms in the dictionary.
- If the candidate plant mention contains both plant name with terms describing specific parts or extracts and its abbreviation, annotators annotate both the plant name excluding part or extract names and the abbreviation term as well. (e.g., *several extracts of* ***Tripterygium wilfordii Hook F (TWHF)***)
- Annotators do not annotate the words that represent part names of plants, for example, roots, stems, and leaves. (e.g., ***persimmon*** *leaf extract, the stem bark of* ***Catalpa ovata***)
- Annotators do not annotate the terms describing the processing methods of plants to extract their active compounds, for example, extraction methods and cooking methods. (e.g., *Korean red* ***ginseng*** *extract, water extracts of* ***Tochu***)
- The plant-based products should not be annotated. (e.g., annotators do not annotate “*chocolate*” made of *cocoa* and “*cigarette*” made of *tobacco*.)
- The plant derived substances should not be annotated. (e.g., annotators do not annotate “*caffeine*,” “*rg3*,” and “*lycopene*” as a plant name.)
- Do not annotate pre-annotated mentions derived from plant names as plants if they do not mean plant themselves. (e.g., “*tobacco mosaic virus*” is not a plant.)
- The scientific name of plants basically consists of a genus name and a specific epithet name, which refers to the species within the genus. The genus name is always mentioned first, followed by the mention of a specific epithet name. Annotators should consider words after a specific epithet’s name (e.g., “*activities of* ***Phryma leptostachya var. asiatica*** *Hara extract*”).

#### Annotation of phenotype mentions

MetaMap with UMLS semantic types is widely utilized in various biomedical NLP tasks for all integrated concepts. As mentioned before, the term “phenotype” refers to any observable characteristic in the human body, including diseases. In this step, we annotated disease names as phenotypes using the deep learning NER model; we also used the results derived with the use of MetaMap that belong to the following UMLS semantic types: T019, T020, T033, T034, T037, T038, T039, T041, T046, T047, T048, T049, T050, T184, T190, and T191. The other names not included in the standard were not used for the annotation. Here, we divided the phenotypes into three categories as follows:

- **Positive phenotype (POS)**: phenotypes with effects that positively affect humans (e.g., *recovery, anti-cancer*, and *anti-inflammatory*).
- **Negative phenotype (NEG)**: phenotypes that are known to be harmful to human health and those that need to be medically healed to suppress the negative effects on humans (e.g., *inflammation, breast cancer*, and *cervical carcinoma*).
- **Neutral phenotype (NEU)**: phenotypes for which there exists an uncertainty regarding their distinguishment into positives or negatives (e.g., *pregnancy, sweating, blood pressure*, and *fat weight*).

After annotating the phenotype mentioned in the article, annotators should consider the category (POS, NEG, or NEU) to which the selected mention belongs according to the definitions presented above. Details regarding the guidelines for the annotation considered for phenotype mentions are described below.

- Annotators manually annotate mentions that satisfy only one of the three categories as the phenotype.
- Annotators should consider words in the form of noun phrases for deciding the scope of entity annotation (e.g., “*acute phase of inflammation*” and “*injured the muscle*” could be associated with “*acute inflammation* [UMLS ID: C0333361]” and “*muscle injury* [UMLS ID: C0410256],” respectively).
- Annotators should annotate the only function of an organism, organ, or tissue as a phenotype mention, not the organism, organ, or tissue itself (e.g., “***liver disorders***” in “*to treat liver disorders*” is the phenotype, but “*liver*” in “*nuclear extracts of the liver*” is not the phenotype).
- Annotators should consider the mention with quantitative concepts like weight, length, or concentration as the phenotype. Although a mention itself is not a phenotype in principle, when this mention appears together with a quantitative concept, it is regarded as a neutral phenotype (e.g., “*body weight*” in “*increase in* ***body weight***,” and “***high blood pressure***” are all deemed phenotypes.)
- If there is a noun phrase containing a phenotype mention, and if it is significantly related to the phenotype, then annotators should annotate all terms in the phrase as the phenotype mention (e.g., the annotator annotates “***oral squamous cell carcinoma***” rather than “*carcinoma*” itself).
- Do not annotate general terms such as “*phenotype*,” “*syndrome*,” “*deficiency*,” and “*complications*.”
- Do not include terms indicating species names like “*human* and *mouse*” (e.g., “to suppress various *human* ***tumors***”).
- If the pre-annotated mention contains the name of a cell line, annotators should annotate it after separating the phenotype mention and cell line name (e.g., in “MDA-MB231 *human breast cancer cell*”, two mentions “***MDA-MB231***” and “***breast cancer***” should be separately annotated).
- Annotators should annotate “any phenotype-induced symptom” as a phenotype, but not use “any chemical-induced symptom” as a phenotype. In other words, annotators should only consider the symptom term present in the mention as a phenotype excluding “chemical-induced.” (e.g., ***diabetes-induced cardiomyopathy*** includes whole words as the phenotype, but in case of “*ethyl phenylpropiolate-induced ear edema*”, “*ear edema*” is only the phenotype mention.)
- Do not annotate simple substances such as “*glucose*” and “*lipid*.”
- The mention of the phenotype model and cell line is considered a negative phenotype. This includes the treatment or assay involving the phenotypes.
- Bacteria and viruses should be considered as a negative phenotype.

#### Annotation of PPRs

Annotators should determine one class label for denoting the relationships between the annotated plants and phenotypes. The class labels for the PPR have been divided into four classes as follows:

- **Increase relationship (Increase)**: A plant-derived compound contextually increases a specific phenotype (e.g., “***Anti-cancer*** *effect of* ***Annona Muricata Linn*** *leaves crude extract* (***AMCE***) *on breast cancer cell line*. [PubMed ID: 27558166]”).
- **Decrease relationship (Decrease)**: A plant-derived compound contextually decreases a specific phenotype (e.g., “*The flowers of* ***Prunus persica Batsch*** *have been used for* ***skin disorders*** *in East Asia from ancient times*. [PubMed ID: 11917253]”).
- **Association relationship (Association)**: A plant-derived compound is contextually related to a specific phenotype. However, it is difficult to define either an increase or decrease (e.g., “*To the best of our knowledge, this is the first description of* ***acute hepatitis*** *associated with* *T. capitatum administration*. [PubMed ID: 12072605]”).
- **Negative relationship (No relation)**: Although a pair of plant and phenotype mentions is observed in the same sentence, there is no relationship between the two mentions. Particularly, the title is always considered as a negative relationship, even if a plant-derived compound is contextually related to a specific phenotype (e.g., “*Differential effects of* ***Viscum album*** *extract IscadorQu on cell cycle progression and apoptosis in* *cancer* cells. [PubMed ID: 15547686]”, “*Anti-obesity* *action of oolong* ***tea***. [PubMed ID: 10094584 (Title)]”). Note that in the released corpus, a negative relationship is not specified because any co-occurrence of plant and phenotype entities without specific relationship types (increase, decrease, and association) can be considered a negative relationship.

### Inter-annotator agreement (IAA) measurement

As the corpus has been manually constructed by the annotators, the quality of corpus data, which is one of the most important issues in the annotation process of the corpus, relies on the knowledge of the annotators. As previously mentioned, the construction of the PPR corpus was organized in the mention-level annotation and relation-level annotation between mentions. Therefore, the inter-annotator agreement (IAA) was independently calculated at each annotation level. In this study, three different IAA measures were calculated to assess the accuracy of the corpus. First, a simple index measurement, defined as the proportion of agreement between the two annotators, is calculated as follows:

1 $$Simple\_index\left({P}_{0}\right)=\frac{number\_of\_agreements}{N},$$

where *N* represents the total number of annotation units. Note that we used the simple index as “Strict matches (Strict)” for full-word matches and “Partial matches (Partial)” for overlap matches. Second, the G-index was used to measure the overall inequality of the annotator’s work and is calculated as follows:

2 $$G\_index=1-\frac{1-{P}_{0}}{1-{P}_{k}},$$

where *P*_0_ represents a simple index, *P*_*k*_ = 1/*k*, and *k* denotes the number of relation classes. Lastly, Cohen’s kappa (*κ*) index^42^ is the most frequently used index for calculating the overall agreement scores between two annotators. The kappa value is calculated as follows:

3 $$Cohen{\prime} s\;\kappa =1-\frac{1-{P}_{0}}{1-{P}_{e}},$$

where *P*_*e*_ represents the hypothetical probability of an agreement by chance. A kappa value of = 1 indicates complete agreement, and kappa = 0 indicates no agreement between the two annotators. According to the study conducted by Viera *et al*.^43^, kappa values ranging from 0.61 to 0.80 denote “substantial” agreement and those presenting with values 0.81 or above indicate “almost perfect” agreement.

#### Disagreements

In the annotation results, we found several fully disagreed cases and partially agreed cases between two annotators. According to our analysis, most of fully disagreed cases occurred in the following cases: (i) one of annotators did not recognize abbreviation for plant mention as plant (ex., SEG = semi-evergreen); (ii) one of annotators mistakenly identified the word, “extract,” which is a substance taken from a plant, as plant name; and (iii) one of annotators annotated terms related to cells, chemical levels, genes as phenotype mentions even though they were not included in the phenotype range (ex., human lung epithelial cells, nitric oxide level, and COX-2). Most of partially agreed cases appeared in the following cases: (i) one of annotators included the words corresponding to plant parts in the plant mention although they should not be included (ex., P. guajava leaf, Persicariae Rhizoma) and (ii) annotators mistakenly regarded chemical-induced disease/symptom or plant-induced disease/symptom as phenotype (ex., Tripterygium wilfordii-induced liver injury, colitis-associated colon cancer, and circulating tumor-related leukocytes).

## Data Records

The PPR corpus is the first corpus annotated with information on plant and phenotype entities and their relationships derived from PubMed abstracts. The PPR corpus consists of data from 600 non-redundant abstracts randomly extracted from the PubMed database, which contains 16,937 mention annotations (with 5,858 unique mentions) and 9,709 relation annotations (with 8,135 unique relations). To facilitate benchmarking experiments, the set of articles must be categorized into train, development, and test sets during corpus construction. We fixed the number of abstracts in development and test sets to 100 by referring to the NCBI disease corpus^19^, as the separated corpus is useful for the development of new algorithms, the avoidance of overfitting, and the accurate evaluation of new models. Therefore, the PPR corpus was divided into 400, 100, and 100 articles as the train, development, and test sets, respectively.

Table 1 shows the overall statistics of entity and relation annotations in the PPR corpus, and the three data sets exhibit similar aspects of the number of mentions and relations, which renders increased utility to the corpus for training models. In addition, we compared overlapping of entities and relations among training, development, and test sets. Here, the overlap between entities means exactly the same in annotated mentions and their types. For the relations, the overlap means that both entities and their relationship have identical annotation. For plant entities, 242 out of 934 (25.91%) mentions in the development set and 180 out of 968 (18.60%) mentions in the test set overlapped with plant entities in the train set. For phenotype entities, 1,249 out of 1,803 (69.27%) mentions in the development set and 1,174 out of 1,812 (64.79%) mentions in the test set overlapped with phenotype entities in the train set. Since we used biomedical literature to build the PPR corpus and specifically defined observable characteristics in a human as a phenotype in this study, the phenotype names overlaps relatively more than plant names. For relationships, 45 out of 1,563 (2.88%) relations in the development set and 25 out of 1,569 (1.59%) relations in the test set overlapped with the relation of the train set. Because the PPR corpus contains various kinds of plant names and plant-phenotype relationships with little redundancies, NLP models trained by the PPR corpus may show robust performances any other data set.

**Table 1:** Overall corpus statistics.

Set	Abstracts	Entity *(# of unique entities)*					Relationships *(# of unique relations)*				
		Plant	Phenotype			Total	Increase	Decrease	Association	Negative (No relation)	Total
			POS	NEG	NEU						
Train	400	3752 (1031)	1702 (410)	5605 (1974)	361 (190)	11420 (3605)	971 (855)	2808 (2424)	52 (49)	2746 (2211)	6577 (5539)
Development	100	934 (281)	466 (166)	1283 (614)	54 (42)	2737 (1103)	265 (239)	608 (519)	13 (13)	677 (511)	1563 (1282)
Test	100	968 (322)	437 (152)	1339 (650)	36 (26)	2780 (1150)	242 (210)	663 (577)	2 (2)	662 (525)	1569 (1314)
Total	600	5654 (1634)	2605 (728)	8227 (3238)	451 (258)	16937 (5858)	1478 (1304)	4079 (3520)	67 (64)	4085 (3247)	9709 (8135)

The PPR corpus consists of training, development, and test sets for plant and phenotype name recognition and relation extraction tasks.

**Fig. 2:**
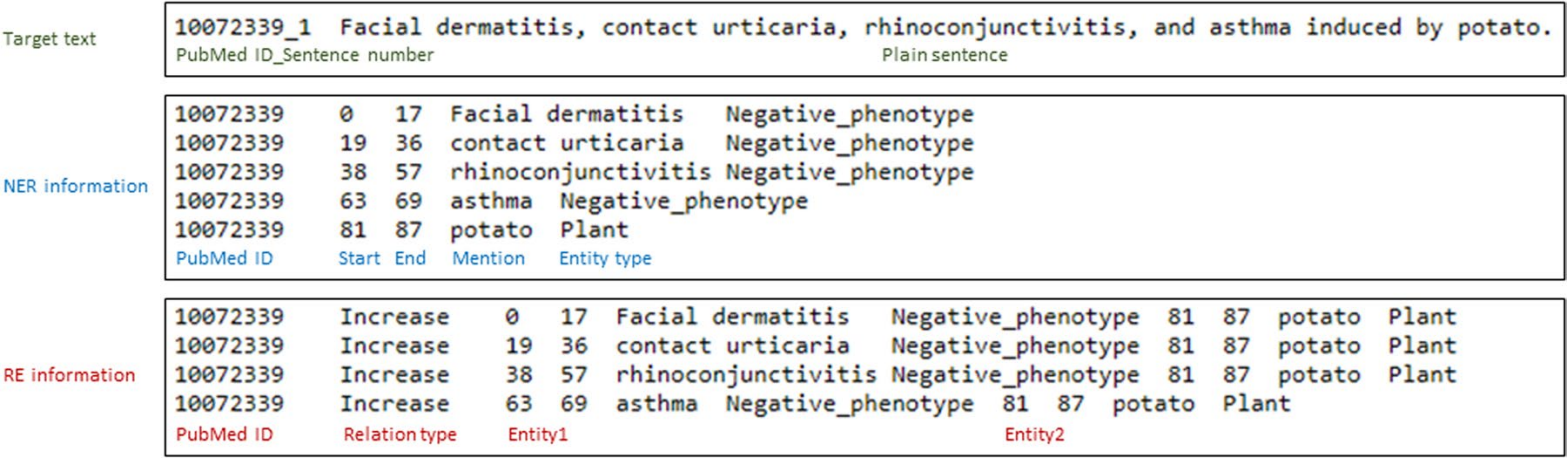
Example of the PPR corpus. The first line is a sentence obtained from the first sentence of an abstract (PubMed ID: 10072339), followed by annotated named entities and their relationships. The named entity information includes PubMed ID, start and end positions, annotated mention, and entity type. The relationship information consists of PubMed ID, relation type, and information related to the two entities.

The PPR corpus contains information regarding all sentences of abstracts and annotations of entities and relationships at the sentence level. Figure 2 shows an example of our PPR corpus. All data fields are formatted in tab-delimited text files. For sentences, the fields consist of PubMed ID (e.g., 10072339) with a sentence number separating the underlined and plain sentence. For entities, the fields are as follows: PubMed ID (e.g., 10072339), start index (e.g., 0), end index (e.g., 17), text (e.g., “*Facial dermatitis*”), and entity type (e.g., “Negative_phenotype”). The fields for relationships are as follows: PubMed ID (e.g., 10072339), relationship type (e.g., “Increase”), front entity information (e.g., “0 17 *Facial dermatitis* Negative_phenotype”), and rear entity information (e.g., “81 87 *potato* Plant”).

The PPR corpus is publicly available at two locations:

- Figshare, an open scientific data repository^44^.
- A git repository of the project is accessible at https://github.com/DMCB-GIST/PPRcorpus.

## Technical Validation

### Inter-annotator agreements

In this annotation task, the construction of the PPR corpus included six phases comprising the inclusion of 100 abstracts per phase, and two annotators who were experienced in biomedical text-mining participated in the experiments. Table 2 describes the overall IAA results of each phase between two annotators for the construction of the PPR corpus. The average “strict matches” IAA scores for plant and phenotype mentions were 91.5% and 66.4%, respectively. When the “partial matches” IAA scores were calculated, two mention-level annotations yielded IAA scores higher than those obtained using “strict matches.” The average “Partial” IAA scores were 94.8% of the plant mentions and were 80.9% of the phenotype mentions, representing higher agreement with the consideration of plant mentions compared to phenotype mentions. The lowest agreement score for each entity is represented in Phase 1 of the phenotype entity annotation and Phase 6 of the plant entity annotation. The annotator disagreements of the phenotype entity in Phase 1 occurred when a few mentions were not present in the annotation guidelines. The conflicts were primarily attributable to the differences in the mention boundaries. We further assessed the IAA scores of the relation-level annotations based on the three criteria considered. Additionally, for relation-level annotations, the average IAA scores according to the simple index, G-index, and Cohen’s kappa were estimated to be 92.2%, 90.7%, and 86.9%, respectively.

**Table 2:** Overall inter-annotator agreement (IAA) results of each phase.

Annotation Phase	Plant		Phenotype		Relations		
	Strict	Partial	Strict	Partial	Simple index	G-index	Cohen’s κ
Phase 1	93.6	96.4	51.5	70.0	90.8	89.7	86.5
Phase 2	93.0	97.0	64.0	93.2	92.5	90.2	85.6
Phase 3	91.7	94.6	64.8	77.6	93.1	91.8	87.2
Phase 4	93.5	95.8	72.4	82.0	92.8	90.3	86.4
Phase 5	92.8	95.2	78.0	86.0	91.9	90.6	87.5
Phase 6	84.4	89.5	67.5	76.3	92.2	91.5	88.3
Average	91.5	94.8	66.4	80.9	92.2	90.7	86.9

The PPR corpus was annotated by two annotators at the mention- and relation-levels. Therefore, IAA was independently calculated at each annotation level, and three different IAA measures were calculated to assess the accuracy of the corpus.

#### IAA for entities

Table 3 shows the overall statistics of the PPR corpus in comparison with the previously published corpora for biomedical NER tasks, grouped by entity types. All mentions in the PPR corpus are annotated as two types of entities, plant and phenotype names, in which 5,668 and 11,282 mentions are mapped, respectively. For plant type, although species corpora (LINNAEUS^24^ and Species-800^25^) contain the names of all organisms, including botanical terminology, the PPR corpus demonstrates the presence of a greater number of plant name annotations. To obtain insights into the diversity of plant names within the corpus, the PPR corpus is constructed based on the information derived from 600 abstracts, which is approximately three times greater than that of the plant corpus^14^. An IAA score of the entity-level was obtained by correctly identifying the mentions classified by each entity type. Although the IAA score of plant mentions in the PPR corpus is inferior to that in the plant corpus^14^ (91.5% vs 98.5%) because of the complicated annotation guideline of the PPR corpus, it also suggests a high level of agreement (“almost perfect” agreement).

**Table 3:** Statistics of the biomedical NER corpora for the annotated entities.

Corpus	Entity type	Text type	# Documents	# Mentions	IAA (%)	IAA metric
2010 i2b2/VA^17^	Clinical concept	Reports	826	72,846	—	—
ShARe/CLEF^18^	Clinical concept	Reports	298	11,167	—	—
NCBI disease^19^	Disease	Abstracts	793	6,892	87.5	Strict
BC5CDR^22^	Disease	Abstracts	1,500	12,850	87.5	Strict
	Chemical	Abstracts	1,500	15,935	96.1	Strict
CHEMDNER^23^	Drug/Chemical	Abstracts	10,000	84,355	91.0	Strict
BC2GM^20^	Gene/Protein	Sentences	20,000	24,596	—	—
JNLPBA^21^	Gene/Protein	Abstracts	2,404	59,963	—	—
LINNAEUS^24^	Species	Full-text	100	4,259	89.0	Cohen’s kappa
Species-800^25^	Species	Abstracts	800	3,708	80.0	Cohen’s kappa
Plant^14^	Plant	Abstracts	208	3,985	98.5	Strict
PPR	Plant	Abstracts	600	5,654	91.5/94.8	Strict/Partial
	Phenotype	Abstracts	600	11,283	66.4/80.9	Strict/Partial

Following the definition in the annotation guideline, it is observed that the phenotype refers to all events, such as positive, negative, and neutral effects on humans. Therefore, the proposed concept of phenotype includes disease and clinical terms. The NCBI disease corpus^19^ comprises 6,892 disease mentions, and the BC5CDR corpus^22^ is composed of 12,850 disease mentions, in which 8.7 mentions and 8.6 mentions per abstract are mapped, respectively. In contrast, clinical reports have a relatively considerable number of clinical term annotations in the corpora. For instance, the 2010 i2b2/VA corpus^17^ comprises 88.2 mentions per report and the ShARe/CLEF^18^ corpus includes 37.5 clinical terms per report. The PPR corpus contains 18.8 phenotype mentions per abstract, which includes more entity information than the published abstract-based disease corpora. The IAA score of phenotype mentions shows low performance compared with others because there were a few changes in the guidelines. However, the final PPR corpus was constructed using a sufficient disagreement resolution process to ensure accuracy.

#### IAA for relations

Table 4 represents the overall statistics of the PPR corpus, together with previously published corpora for biomedical RE tasks, grouped by relation types. The PPR corpus consists of 9,709 annotated PPRs in 600 PubMed abstracts and includes 16.2 relations per abstract. Although CHEMPROT^28^ presents with a similar number of relations with the PPR corpus, it exhibits the presence of two relationships per abstract. Thus, the information in each abstract may not be sufficient. The IAA score of the plant-disease corpus^33^ is similar to that of the PPR corpus; however, its ratio of relations per abstract is 6.6, which is relatively lower than that of the PPR corpus. The plant-chemical corpus^13^ is a sentence unit corpus with only one relation type “contain” used for describing that a plant contains chemicals. Other corpora related to diseases, disorders, and clinical terms, which are also a component of the phenotype, are shown in Table 4, including BC5CDR^22^, EU-ADR^30^, GAD^31^, CoMAGC^32^, and plant-disease^33^. The PPR corpus includes a sufficient number of relations compared to these corpora, and the IAA result of the PPR corpus showed “almost perfect” agreement (kappa = 0.869). Moreover, a ratio of relations per abstract of the PPR corpus is as high as 16.2 relations while that of the plant-disease corpus is 6.6.

**Table 4:** Statistics of the biomedical RE corpora for the annotated relationships.

Corpus	Relation type	Text type	# Documents	# Relations	IAA (%)	IAA metric
AIMed^26^	Protein-Protein	Sentences	1,955	5,834	—	—
BioInfer^27^	Protein-Protein	Sentences	1,100	9,666	—	—
BC5CDR^22^	Chemical Disease	Abstracts	1,500	3,116	—	—
CHEMPROT^28^	Chemical-Protein	Abstracts	5,031	10,031	—	—
DDI^29^	Drug-Drug (Drugbank)	Documents	792	4,701	83.9	Cohen’s kappa
	Drug-Drug (PubMed)	Abstracts	233	327	62.1	Cohen’s kappa
EU-ADR^30^	Drug-Disorder	Abstracts	100	668	73.3	(relaxed) Simple index
	Target-Disorder	Abstracts	100	941	74.0	(relaxed) Simple index
	Target-Drug	Abstracts	100	827	75.7	(relaxed) Simple index
GAD^31^	Gene-Disease	Sentences	5,330	5,330	—	—
CoMAGC^32^	Gene-Cancer	Abstracts	408	821	75.7/56.8	Cohen’s kappa
Plant-Disease^33^	Plant-Disease	Abstracts	199	1,309	86.9	Cohen’s kappa
Plant-Chemical^13^	Plant-Chemical	Sentences	382	1,043	79.8	Cohen’s kappa
PPR	Plant-Phenotype	Abstracts	600	9,709	86.9	Cohen’s kappa

### The evaluation techniques

As an application of the PPR corpus, we used NER and performed relation extraction (RE) tasks to the corpus. NER is one of the most widely known text mining-related tasks, which involves recognition of numerous domain-specific entities in the biomedical text, and RE is another commonly studied NLP task to classify relationships between the recognized named entities in text. For the NLP tasks with the best performance, most researchers previously used various combinations of hidden layers such as deep neural networks and conditional random fields architectures^10,11^. Recently established deep learning methods, especially contextualized language models such as BERT^12^, have resulted in significant improvements in many NLP tasks, including NER and RE. Therefore, we considered fine-tuning BERT-based models such as BERT^12^, BioBERT^45^, BlueBERT^46^, SciBERT^47^, and PubMedBERT^48^.

- **BERT**^12^: BERT is a contextual language representation model using pre-training deep bidirectional representations from unlabeled text. Instead of conducting traditional left-to-right language modeling, BERT is trained on two tasks: a masked language model (MLM) by predicting randomly masked tokens and a next sentence prediction (NSP) by predicting whether two sentences follow each other. BERT demonstrates a simple architecture based on the transformer and shows powerful performance in various NLP tasks, while illustrating the potential of the fine-tuning approach.
- **BioBERT**^45^: BioBERT is a domain-specific language representation model designed for biomedical text and is initialized with the checkpoint of BERT, followed by training of the BERT model using PubMed abstracts and PubMed Central full-text articles. BioBERT achieves SOTA performance in various biomedical NLP tasks with minimal task-specific fine-tuning, while requiring only minimal architectural modifications.
- **BlueBERT**^46^: Similar to BioBERT, BlueBERT is recognized as another variant of BERT, which is initialized with BERT and is further pre-trained using information available in PubMed abstracts and clinical notes derived from MIMIC-III. The standard approach of utilization of BERT-based models, such as BioBERT, is initialized with application of the BERT model, followed by continuous conduction of the pre-training process with MLM and NSP using their respective corpora.
- **SciBERT**^47^: SciBERT is a variant of BERT-based models and demonstrates the same architecture as that exhibited by BERT. While BERT was pre-trained using general-domain corpora, SciBERT was pre-trained using information available in a greater number of scientific papers that consist of complete textual content based on computer science and biomedical domains. Previously, the vocabulary was considered the same as the original BERT model generated using information available in Wikipedia and BookCorpus. A major disadvantage of this approach is that vocabulary is not representative of the target biomedical domain. Therefore, they constructed a new in-domain vocabulary for their scientific text corpora, called SciVocab, to overcome the problem of the continual pre-training approach.
- **PubMedBERT**^48^: PubMedBERT is another pre-trained language model exhibiting the same architecture as that demonstrated by BERT. Unlike those observed in the mixed-domain pre-training models, the weights of the PubMedBERT model were not initialized with those of BERT during pre-training. They constructed an in-domain vocabulary of the target biomedical domain and pre-trained it using information available in PubMed abstracts and additional data in full-text PubMed Central articles. The PubMedBERT model was pre-trained using information available in PubMed abstracts and additional data in full-text PubMed Central articles, which comprised data derived from 14 million PubMed abstracts with 3 billion words and contained 21 GB of textual data in total.

**Fig. 3:**
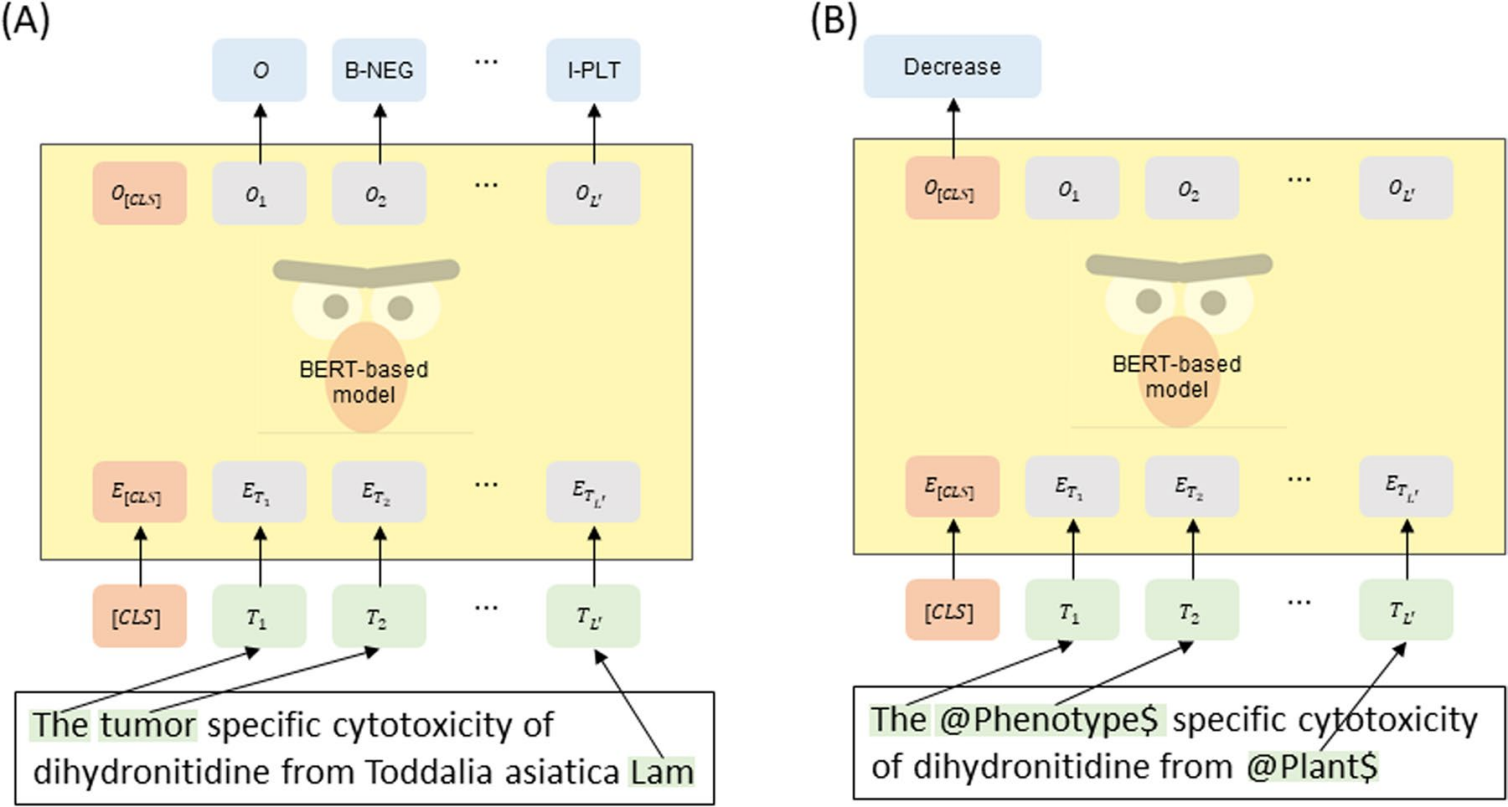
BERT-based fine-tuning model architectures. The input sentence is “The *tumor* specific cytotoxicity of dihydronitidine from *Toddalia asiatica Lam* (PubMed ID: 16465544).” In this case, “tumor” is annotated with the negative phenotype, and “Toddalia asiatica Lam” is the plant mention. Figure (**A**) represents the BERT-based NER model, and Figure (**B**) shows the BERT-based RE model.

Using the PPR corpus, we used five types of BERT-based models for biomedical NER and RE tasks between plant and phenotype entities and compared the performance of pre-trained models. For fair comparison, all parameters of the BERT-based models are set to the default values described in BERT^12^. Figure 3 illustrates the fine-tuning techniques of NER and RE using the BERT-based models. In the NER task, the NER process involves the classification of entities with proper boundaries and types based on informal texts. To recognize the entity in texts, each token in the input sentence is assigned the BERT-based classifier, and the label of each token is determined through the probabilities calculated by using the SoftMax function. In the biomedical domain, entity names are usually extremely complex and specific; hence, the vocabulary of models must contain all tokens in sentences. The anonymization process prevents the RE model from exhibiting bias toward specific words or tokens. For simplicity, we anonymized target entities in a sentence by replacing the plant entity with “@Plant$” and the phenotype entity with “@Phenotype$.” As the RE task is usually considered as a classification issue at the sentence or sequence level, the achievement of a representation of a certain number of dimensions for the input sequence is necessary. We presented the sentence using the “[CLS]” token of BERT’s last hidden layer despite common practice^49^. Particularly, the embeddings of the “[CLS]” token typically act as pooling token embeddings representing the whole sequence for downstream tasks. For the RE task, the label of the input sentence is decided based on the probabilities of relation classes using the SoftMax function.

Our evaluation metrics were micro-F1, macro-F1, and weighted-F1 scores. In such cases, precision(p) was defined as the number of true positives divided by the number of predictions, recall(r) was defined as the number of true positives divided by the number of annotations, and F1-score(f) was defined as the harmonic mean of precision and recall. Micro-F1 score is used to enumerate the global true positives, false positives, and false negatives, whereas macro-F1 is used to convey the average unweighted class scores. Since macro-F1 is often used to assign equal weights to both frequent and infrequent classes, we must consider the entity type distribution. Weighted-F1 scores are used to denote the average “weighted” class scores to consider class imbalance in the PPR corpus.

### Named entity recognition

For named entity recognition, we evaluate performance via strict matching, which helps to evaluate both the boundary and entity types of mentions. Table 5 shows the comparison of NER performance for precisions, recalls, and F-scores of the five BERT-based models which consist of two types of evaluation experiments. The first approach is to use a divided corpus where the training set is used to train a model, the development set is used to optimize the model during training time, and the test set is used to evaluate the performance of the model. Since we always use the same data for evaluation under all cases, this method can be used to evaluate the benchmark system. The second approach is the well-known *k*-fold cross-validation method. *k*-fold cross-validation is an objective approach for examining the accuracy of statistical prediction methods. Thus, there is no need to artificially separate the corpus into train and test sets. In this study, we used the method of 5-fold cross-validation (*k* = 5). In the PPR test set, although BioBERT demonstrated the obtainment of a relatively lower macro recall than that obtained by using PubMedBERT (86.80% vs. 87.07%), BioBERT exhibited the achievement of the best F1 scores for all types of evaluations. In the 5-fold cross-validation method, the fine-tuned BioBERT outperformed all other models.

**Table 5:** Evaluation of the BERT fine-tuned models to recognize plant and phenotype mentions based on the conduction of two types of evaluation experiments.

Model	Test (100 abstracts)									5-fold cross validation								
	micro-F1			macro-F1			weighted-F1			micro-F1			macro-F1			weighted-F1		
	p	r	f	p	r	f	p	r	f	p	r	f	p	r	f	p	r	f
BERT	81.25	85.58	83.36	74.82	83.61	78.55	81.46	85.58	83.43	82.03	84.57	83.28	77.78	80.33	78.99	82.06	84.57	83.28
BioBERT	**87**.**49**	**91**.**04**	**89**.**23**	**78**.**66**	86.80	**82**.**11**	**87**.**70**	**91**.**04**	**89**.**31**	**87**.**82**	**89**.**98**	**88**.**89**	**81**.**79**	**84**.**36**	**83**.**01**	**87**.**86**	**89**.**98**	**88**.**90**
BlueBERT	82.54	86.87	84.65	73.80	83.70	77.72	82.90	86.87	84.79	83.44	86.46	84.92	72.57	75.71	74.07	83.51	86.46	84.95
SciBERT	84.13	88.13	86.09	74.38	83.01	77.92	84.48	88.13	86.23	85.26	87.64	86.43	77.71	79.91	78.75	85.28	87.64	86.43
PubMedBERT	82.84	87.70	85.20	74.89	**87**.**07**	79.55	83.23	87.70	85.33	83.90	87.08	85.46	79.40	82.79	81.00	83.96	87.08	85.48

Table 6 represents performance comparison of BERT-based models based on the target entity types used to divide plant and phenotype names: ALL, PLT, PHE, PLT/PHE, and POS/NEG/NEU. Originally, the PPR corpus consisted of four types of plants and three subtypes of phenotypes (negative, positive, and neutral), as “ALL” in Table 6. The model of “PLT” considers only plant mentions. The “POS/NEG/NEU” model recognizes only phenotype mentions such as positive, negative, and neutral phenotypes, respectively, whereas the models of “PHE” recognize the only phenotype mentions regardless of the subtypes of phenotype mentions. The model of “PLT/PHE” is used simultaneously with two types of named entities as plant and phenotype mentions regardless of the subtypes. Similar to Table 5, BioBERT in Table 6 shows outperformed performance with respect to all cases except for the macro recall. Moreover, BioBERT outperformed other types of ALL, PHE, PLT/PHE, and POS/NEG/NEU.

**Table 6:** Performance comparison of BERT-based models based on target entity types used to divide plant and phenotype names: a total of four types for plants and three subtypes of phenotypes (ALL), only plant (PLT), the only phenotype (PHE), two types of named entities as plant and phenotype mentions regardless of the subtypes (PLT/PHE), and only phenotype mentions such as positive, negative, neutral phenotypes (POS/NEG/NEU).

Model		macro-F1			macro-F1			weighted-F1		
		p	r	f	p	r	f	p	r	f
BERT	All	81.25	85.58	83.36	74.82	83.61	78.55	81.46	85.58	83.43
	PLT	82.36	84.40	83.37	82.36	84.40	83.37	82.36	84.40	83.37
	PHE	81.89	84.60	83.22	81.89	84.60	83.22	81.89	84.60	83.22
	PLT/PHE	81.59	85.90	83.69	81.70	86.44	84.00	81.58	85.90	83.68
	POS/NEG/NEU	81.14	84.27	82.67	71.15	81.33	75.23	81.54	84.27	82.83
BioBERT	All	**87**.**49**	**91**.**04**	**89**.**23**	**78**.**66**	86.80	**82**.**11**	**87**.**70**	**91**.**04**	**89**.**31**
	PLT	**87**.**76**	**92**.**56**	**90**.**10**	**87**.**76**	**92**.**56**	**90**.**10**	**87**.**76**	**92**.**56**	**90**.**10**
	PHE	**86**.**67**	**88**.**96**	**87**.**80**	**86**.**67**	**88**.**96**	**87**.**80**	**86**.**67**	**88**.**96**	**87**.**80**
	PLT/PHE	**88**.**81**	**91**.**04**	**89**.**91**	**89**.**58**	**91**.**85**	**90**.**70**	**88**.**81**	**91**.**04**	**89**.**91**
	POS/NEG/NEU	**86**.**83**	**89**.**90**	**88**.**34**	**76**.**13**	**85**.**59**	**80**.**08**	**87**.**15**	**89**.**90**	**88**.**46**
BlueBERT	All	82.54	86.87	84.65	73.80	83.70	77.72	82.90	86.87	84.79
	PLT	83.98	89.36	86.59	83.98	89.36	86.59	83.98	89.36	86.59
	PHE	84.24	87.64	85.91	84.24	87.64	85.91	84.24	87.64	85.91
	PLT/PHE	83.55	87.30	85.38	83.82	87.83	85.78	83.54	87.30	85.38
	POS/NEG/NEU	83.02	86.09	84.53	71.84	80.60	75.41	83.44	86.09	84.70
SciBERT	All	84.13	88.13	86.09	74.38	83.01	77.92	84.48	88.13	86.23
	PLT	85.91	89.46	87.65	85.91	89.46	87.65	85.91	89.46	87.65
	PHE	84.51	88.25	86.34	84.51	88.25	86.34	84.51	88.25	86.34
	PLT/PHE	84.79	89.21	86.94	84.78	89.72	87.18	84.79	89.21	86.93
	POS/NEG/NEU	84.86	87.86	86.33	73.95	83.46	77.89	85.20	87.86	86.47
PubMedBERT	All	82.84	87.70	85.20	74.89	**87**.**07**	79.55	83.23	87.70	85.33
	PLT	83.14	88.64	85.80	83.14	88.64	85.80	83.14	88.64	85.80
	PHE	83.45	86.53	84.96	83.45	86.53	84.96	83.45	86.53	84.96
	PLT/PHE	83.12	87.70	85.35	83.12	87.99	85.48	83.12	87.70	85.35
	POS/NEG/NEU	83.27	84.88	84.07	74.78	82.50	78.01	83.53	84.88	84.16

### Relation information extraction

For relation information extraction, we also performed experiments to elucidate RE performance using five different BERT-based models. Table 7 demonstrates the results of each RE model shown in the same manner using the NER evaluation. In the PPR test set, the fine-tuned PubMedBERT demonstrated the obtainment of the best micro-, macro-, and weighted F-scores compared with the other models. After PubMedBERT, BioBERT showed the second-best performance. For a more detailed comparison, we also performed 5-fold cross-validation in the RE task. In the 5-fold cross-validation, although BlueBERT demonstrated the achievement of a higher micro F-score (87.50%) than the others, PubMedBERT showed the achievement of best macro and weighted F-scores of 68.66% and 87.34%, respectively. Since the PPR corpus contains data on a relatively small number of association relations, the macro F-score seems to be lower than the micro-and weighted F-scores. In conclusion, although BioBERT showed the best performance in the NER task, PubMedBERT generally showed the best performance in the RE task.

**Table 7:** Evaluation of BERT fine-tuned models to extract information on the relationships between plant and phenotype mentions based on the conduction of two types of evaluation experiments.

Model	Test (100 abstracts)									5-fold cross validation								
	micro-F1			macro-F1			weighted-F1			micro-F1			macro-F1			weighted-F1		
	p	r	f	p	r	f	p	r	f	p	r	f	p	r	f	p	r	f
BERT	83.88	83.88	83.88	62.70	61.46	61.97	84.16	83.88	83.88	85.97	85.97	85.97	67.60	65.93	66.20	86.08	85.97	85.84
BioBERT	87.83	87.83	87.83	65.41	65.57	65.44	87.83	87.83	87.75	87.16	87.16	87.16	68.38	66.69	67.05	87.24	87.16	86.99
BlueBERT	86.55	86.55	86.55	64.69	64.29	64.42	86.66	86.55	86.49	**87**.**50**	**87**.**50**	**87**.**50**	67.06	66.41	66.52	87.52	**87**.**50**	87.33
SciBERT	83.88	83.88	83.88	62.50	61.59	61.97	84.07	83.88	83.86	86.19	86.19	86.19	67.56	65.87	66.26	86.34	86.19	86.05
PubMedBERT	**88**.**78**	**88**.**78**	**88**.**78**	**66**.**02**	**66**.**73**	**66**.**28**	**89**.**07**	**88**.**78**	**88**.**80**	87.48	87.48	87.48	**71**.**45**	**68**.**01**	**68**.**66**	**87**.**63**	87.48	**87**.**34**

## Usage Notes

The PPR corpus is made available under the Creative Commons Attribution 4.0 International Public License (CC-BY). The codes to run this corpus is available at https://github.com/DMCB-GIST/PPRcorpus. Compared to other plant-disease corpora, the PPR corpus defined positive and negative phenotypes, which is a wide range of characteristics observable in a human, so that it can explain a wider range about medical information than the plant-disease corpus. Negative phenotype is known to be harmful to human health and need to be medically healed to suppress the negative effects on humans. Thus, disease name in our corpus is a part of the negative phenotypes.

## Data Availability

The Python codes for the NER and RE experiments represented in Technical Validation can be accessed from https://github.com/DMCB-GIST/PPRcorpus.
